# Modular Supervisory Control for the Coordination of a Manufacturing Cell with Observable Faults

**DOI:** 10.3390/s23010163

**Published:** 2022-12-23

**Authors:** Nikolaos D. Kouvakas, Fotis N. Koumboulis, Dimitrios G. Fragkoulis, Aristotelis Souliotis

**Affiliations:** 1 Department of Digital Industry Technologies, School of Science, National and Kapodistrian University of Athens, Euripus Campus, 34400 Evia, Greece; 2 Core Department, National and Kapodistrian University of Athens, Euripus Campus, 34400 Evia, Greece

**Keywords:** industrial processes, discrete event systems, supervisory control, fault tolerant control

## Abstract

In the present paper, a manufacturing cell in the presence of faults, coming from the devices of the process, is considered. The modular modeling of the subsystems of the cell is accomplished using of appropriate finite deterministic automata. The desired functionality of the cell as well as appropriate safety specifications are formulated as eleven desired languages. The desired languages are expressed as regular expressions in analytic forms. The languages are realized in the form of appropriate general type supervisor forms. Using these forms, a modular supervisory design scheme is accomplished providing satisfactory performance in the presence of faults as well guaranteeing the safety requirements. The aim of the present supervisor control scheme is to achieve tolerance of basic characteristics of the process coordination to upper-level faults, despite the presence of low-level faults in the devices of the process. The complexity of the supervisor scheme is computed.

## 1. Introduction

In flexible manufacturing systems (FMS), the infrastructure is composed of basic components (robots, computer numerical control (CNC), assembling machines, and storing systems) or islands of components, where each island of components is unreconfigurable. This consideration requires a two-layer control design. In the first layer, the components and/or the islands of components are controlled using the respective local sensors and actuators. The control objective of this layer is to perform specific activities of the subsystem [1]. In the second layer, the control objective is the synchronization/coordination of the individual subsystems to satisfy safety and functionality specifications of the overall manufacturing process [1]. The flexibility of the process results from modification of the second layer controller. Advances in the controller hardware contribute toward this scheme see [2,3]. However, as the Programmable Logic Controllers (PLCs) is one of the main architectures of manufacturing system control the use of formal methods for controller synthesis and PLC program design (with standardized languages, e.g., International Electrotechnical Commission (IEC) 61131-3) is crucial, see [2,3,4,5]. Supervisory control theory (SCT) [6] is a formal method that tackles the above problems crossing the bridge between the event-based automation and the synchronous signal-based PLC world, see [1,7]. Most commonly, Ladder Diagrams (for PLCs) are used to implement monolithic or modular supervisors. For the definition of monolithic and modular supervisors, see [6,8]. Monolithic supervisors suffer from state explosion as the models grow. The implementation of a single supervisor with many states could make the control program unreliable and/or even unstable [1]. Modular supervisor design requires on-the-fly synchronization of the plant and the controller [5]. However, it reduces computational complexity by reducing the total number of states [1].

The supervisory control design in the presence of faults of the manufacturing process is of particular importance. Indicatively, see [9,10] and the references therein. In the present paper, it is considered that the sensors and the actuators can fail in any time, and once a sensor fault took place, the fault is permanent and requires repair, see also [10]. Here, a modular modelling and supervisor design method will be applied to a manufacturing cell presented in [1,3,11]. The method is in the Ramadge Wonham framework, see [6,8] and the references therein, being one of the milestones of SCT. The proposed supervisor design is based on the consideration of real time knowledge of the occurrence of faults, see [12], through appropriate sensors and/or diagnosers that accomplish the detection and/or isolation of faults. Regarding fault diagnosis, many methods has been developed. Indicatively, see [13] as well [14,15,16], where diagnosis methods using discrete event systems (DES) are presented.

In the present paper, a fault driven supervisory control scheme will be designed considering that the faults are a priori defined and are observable by appropriate monitoring systems. The presence of faults may cause discoordination of the process or even damage of devices. For instance, drilling tool wear or tool brake may damage the manufactured product. Also, a fault in the robotic manipulator may cause product overflow on the table. Furthermore, faults in the circular rotating table can cause a material overflow or a material underflow in the cell’s components. Such faults may cause serious damages in the manufacturing cell’s devices and/or the manufactured products. In general, the presence of faults in the devices of the manufacturing process may cause discoordination of the process, being an upper-level fault of composite type. The present supervisory control scheme aims towards achieving fault tolerance in basic characteristics of the coordination of the process, despite the presence of lower-level faults, namely technical faults in the devices of the process. Thus, the proposed supervisory control scheme guarantees the safety of the system despite the presence of lower-level faults. All unwanted series of actions, that may cause system discoordination, are avoided despite the presence of faults. 

According to [17], in modern automation systems, fault handling gains more and more attention. Clearly, an early fault detection and fault treatment may eliminate the undesired sequences caused by the fault. The main motivation of the present work is the avoidance of the malfunctions, usually met in manufacturing of the present type, by forcing the controlled system to stop its evolution before the execution of the undesired sequences, see also [17,18]. The design of a supervisory control scheme for a well-established and experimentally tested manufacturing cell, with many applications (indicatively see [1,3,11]) is another motivation of the present research. Also, for the manufacturing cell at hand, several supervisors, have been implemented in PLC environment and tested, indicatively see [1,3,11]. The above characteristics contribute to the feasibility of the proposed safety oriented supervisory control scheme.

The first contribution of the paper is the modelling of the manufacturing cell in the presence of faults in every subsystem. The second is the presentation of the system’s specifications in the form of regular languages, in analytic forms, and the realization of the supervisors controlling the process. It is mentioned that most of the supervisors are realized through general prespecified supervisor forms that facilitate the proof of controllability and nonblocking, as well as PLC implementation. The third contribution of the present paper is the implementation of the proposed supervisor using Ladder Diagrams and function blocks. The final contribution of the paper is the distributed analysis in both modeling and nonblocking supervisory design of the manufacturing cell, providing a clear and sufficient method for future case studies of the present type.

The proposed supervisors have been designed to be as possible maximally permissive, while they guarantee the PR property of the supervisors regarding the total automaton of the manufacturing cell. To accomplish this, all uncontrollable events of a supervisor are required to belong to the active event sets of all supervisor’s states. A useful direction is to use the active event set based criteria for PR of the supervisors to check the controllability property of the desired languages. Another useful direction is the use of the active event sets to facilitate the proof of the controllability and the nonblocking property. Regarding system modelling, the decomposition of the system to subsystems appears to facilitate fault modeling. Regarding fault modeling, it is important to mention that is a more complete modeling of the system, even if the faults are not detectable. The above direction is becoming more and more necessary in industry 4.0 manufacturing systems, not only because faults affect the productivity and safety of the system but also due to the strong correlation and common control strategies required for fault detection, fault tolerance control, and handling of cyber-attacks, indicatively see [19,20].

## 2. A DES Model of the Manufacturing Cell

### 2.1. Description of the Manufacturing Cell

**Figure 1:**
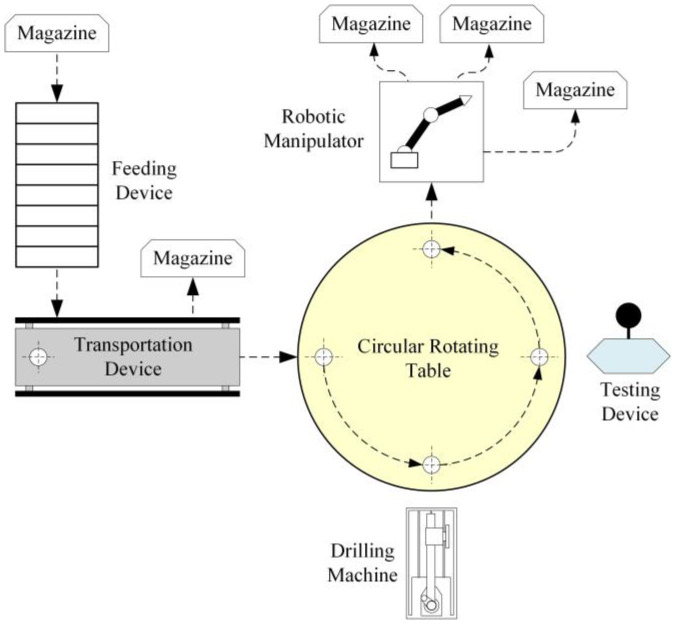
The manufacturing cell.

The manufacturing cell studied here is the cell presented in [1,3,11]. The main components (see Figure 1), namely the subsystems, of the manufacturing cell, are a circular rotating table with four discrete positions, a classifier and transportation device, a drilling machine, a testing device, a robotic manipulator, and a feeding device. The feeding device receives raw products and delivers them to the classifier and transportation (C&T) device. There are three types of raw products, having variable dimensions, that can be transferred into the system. The C&T device classifies the raw products and either transfers them to the table or rejects them. A product is rejected if its dimensions are out of range. The raw products are drilled by the drilling machine and tested by the testing device. The testing device has two different types of processing. Testing process *A* is performed whenever a product has been successfully drilled. Testing process *B* is performed when there is a fault in the drilling process, e.g., tool break down. The products are retrieved from the table by the robotic manipulator. The retrieved products are stored by the manipulator to an appropriate storing magazine. There are three storing magazines. The first type of drilled products are stored to the first magazine. The second type are stored to the second magazine. The rejected drilled products are stored to the third magazine. 

In [1,3,11], the subsystems of the manufacturing cell are presented without considering the presence of faults, except the drilling subsystem where the possibility of the presence of fault has been considered. Here, the possibility of the presence of faults, in all subsystems, is considered. Also here, each subsystem of the manufacturing cell with possible faults will be modelled, in the form of discrete event systems (DES) in the class of finite deterministic automata (see [21,22,23]), i.e., in six tuples of the form $G=(\mathbb{Q},E,f,ℍ,x_{0},{\mathbb{Q}}_{m})$. ℚ denotes the set of the states of G. E denotes the event set (alphabet) of G. ℍ denotes the map from each state of G to the respective set of active events. f denotes the transition function of G. $x_{0}$ denotes the initial state of G. ${\mathbb{Q}}_{m}$ denotes the set of the marked states of G. The closed and the marked behavior of G (see [10]) are denoted by L(G) and $L_{m}(G)$, respectively. For the two behaviors of G, it holds that $L_{m}(G)\subseteq L(G)\subseteq E^{*}$, where $E^{*}$ denotes the Kleene Star of E, see [6,8]. The set of the uncontrollable events of each subsystem is denoted in the form: $E_{uc}\subseteq E$. According to [6,8], if $\bar{L_{m}(G)}=L(G)$ then G is a nonblocking automaton, where $\bar{\cdot }$ denotes the prefix closure of the argument language, see [6,8].

### 2.2. The Model of the Circular Rotating Table with Faults

The model of the circular rotating table in the presence of faults is developed here to be $G_{T}=({\mathbb{Q}}_{T},E_{T},f_{T},{ℍ}_{T},x_{T,0},{\mathbb{Q}}_{T,m})$. The set of the states is ${\mathbb{Q}}_{T}=\{q_{T,1},q_{T,2},q_{T,3}\}$. The initial state is $x_{T,0}=q_{T,1}$. The set of the marked states is ${\mathbb{Q}}_{T,m}=\{q_{T,1}\}$. The states of the circular rotating table are presented in Table 1. The rotating table is in faulty mode when the rotation mechanism is out of order or malfunctions as well as when the rotation is obstructed by obstacles in the workspace. 

The alphabet is $E_{T}=\{e_{T,1},e_{T,2},e_{T,3},e_{T,4}\}$. In Table 2, the events of the circular rotating table, are presented.

The 1st event is a command signal. The 2nd event is a measurable signal. The 3rd event is observable, via an appropriate data acquisition and monitoring system, see [14,15,16]. Clearly, the 1st and the 4th event (repair signal) being produced by the Supervisory Control and Data Acquisition (SCADA) or a button pushed on by the supervising/maintenance personnel, are observable. The set of the controllable events is $E_{T,c}=\{e_{T,1},e_{T,4}\}$ and the set of the uncontrollable events is $E_{T,uc}=\{e_{T,2},e_{T,3}\}$. The sets of the active events are ${ℍ}_{T}(q_{T,1})=\{e_{T,1}\}$, ${ℍ}_{T}(q_{T,2})=\{e_{T,2},e_{T,3}\}$ and ${ℍ}_{T}(q_{T,3})=\{e_{T,4}\}$. The values of the transition functions are $f_{T}(q_{T,1},e_{T,1})=q_{T,2}$, $f_{T}(q_{T,2},e_{T,2})=q_{T,1}$, $f_{T}(q_{T,2},e_{T,3})=q_{T,3}$ and $f_{T}(q_{T,3},e_{T,4})=q_{T,1}$.

**Figure 2:**
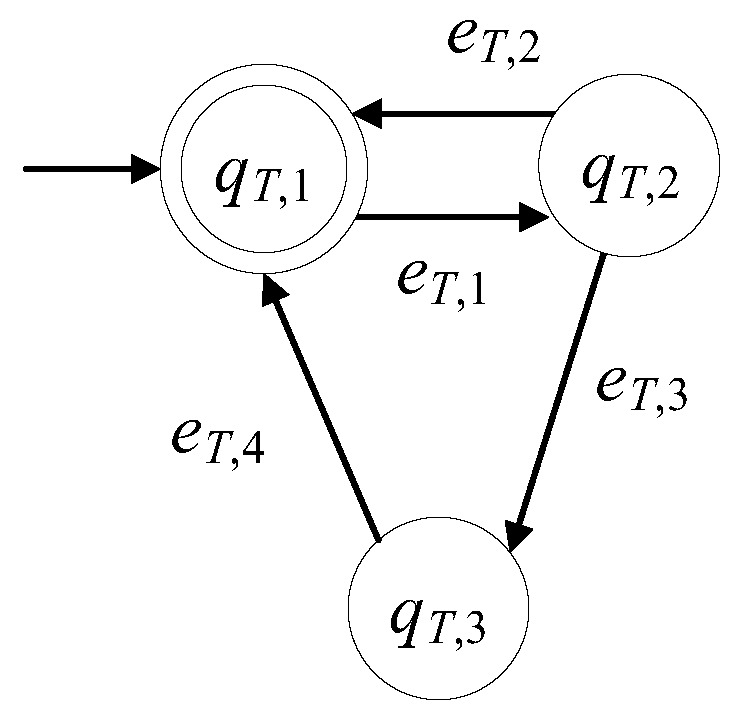
The state diagram of $G_{T}$.

$G_{T}$ is a nonblocking automaton, i.e., $L(G_{T})=\bar{L_{m}(G_{T})}$, where $L_{m}(G_{T})={\left(e_{T,1}(e_{T,2}+e_{T,3}e_{T,4})\right)}^{*}$. In Figure 2, the state diagram of $G_{T}$ is presented. If the presence of faults is neglected, then the state diagram is reduced to that in, see [1,3,11]. 

### 2.3. The Model of the Classifier and Transportation Device with Faults

The model of the C&T device, in the presence of faults, is developed here to be $G_{C}=({\mathbb{Q}}_{C},E_{C},f_{C},{ℍ}_{C},x_{C,0},{\mathbb{Q}}_{C,m})$. The set of the states is ${\mathbb{Q}}_{C}=\{q_{C,1},q_{C,2},q_{C,3},q_{C,4},q_{C,5}\}$. The initial state is $x_{C,0}=q_{C,1}$. The set of the marked states is ${\mathbb{Q}}_{C,m}=\{q_{C,1}\}$. In Table 3, the states of the C&T device are presented. According to [11] the C&T device consist of two linear actuators, a capacitive sensor, an optic sensor, an inductive sensor and an appropriate sensor for the height measurement of the pieces. Regarding the linear actuator, the C&T device is in faulty mode due to an excess of wear, a cracking, a backlash, lubricant related faults, etc., see [24]. Regarding the sensors, the C&T device is in faulty mode due to an external interference to the measurements, very common short-circuit faults, and common sensor drift, see [25].

The alphabet is $E_{C}=\{e_{C,1},e_{C,2},e_{C,3},e_{C,4},e_{C,5},e_{C,6},e_{C,7}\}$. In Table 4, the events of the C&T device are presented.

The events $e_{C,1}$ and $e_{C,4}$ are command signals, and the events $e_{C,2}$, $e_{C,3}$, and $e_{C,5}$ are measurable signals. The event $e_{C,6}$ is observable through an appropriate monitoring system, see [14,15,16]. Clearly, the two command signals and the repair signal, being produced by the SCADA or a button pushed by the supervising/maintenance personnel, are observable. The controllable event set is $E_{C,c}=\{e_{C,1},e_{C,4},e_{C,7}\}$ and the uncontrollable event set is $E_{C,uc}=\{e_{C,2},e_{C,3},e_{C,5},e_{C,6}\}$. The sets of the active events of $G_{C}$ are


$${ℍ}_{C}(q_{C,1})=\{e_{C,1}\},\;{ℍ}_{C}(q_{C,2})=\{e_{C,2},e_{C,3},e_{C,6}\},\;{ℍ}_{C}(q_{C,3})=\{e_{C,4},e_{C,6}\},\;{ℍ}_{C}(q_{C,4})=\{e_{C,5},e_{C,6}\},\;{ℍ}_{C}(q_{C,5})=\{e_{C,7}\}.$$

The values of the transition function of $G_{C}$ are 


$$f_{C}(q_{C,1},e_{C,1})=q_{C,2},\;f_{C}(q_{C,2},e_{C,2})=q_{C,3},\;f_{C}(q_{C,2},e_{C,6})=q_{C,5},\;f_{C}(q_{C,2},e_{C,3})=q_{C,1},\;f_{C}(q_{C,3},e_{C,4})=q_{C,4},\;f_{C}(q_{C,3},e_{C,6})=q_{C,5},\;f_{C}(q_{C,4},e_{C,5})=q_{C,1},\;f_{C}(q_{C,4},e_{C,6})=q_{C,5},\;f_{C}(q_{C,5},e_{C,7})=q_{C,1}.$$

$G_{C}$ is a nonblocking automaton, i.e., $\bar{L_{m}(G_{C})}=L(G_{C})$, where

$$L_{m}(G_{C})={\left(e_{C,1}\left(e_{C,3}+e_{C,6}e_{C,7}+e_{C,2}\left(e_{C,6}e_{C,7}+e_{C,4}(e_{C,5}+e_{C,6}e_{C,7})\right)\right)\right)}^{*}.$$

**Figure 3:**
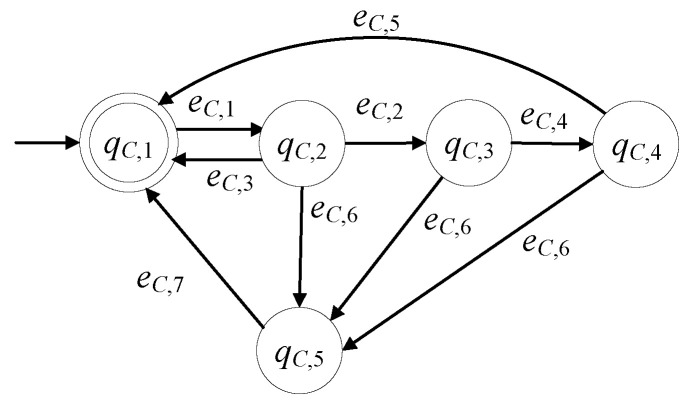
State diagram of $G_{C}$.

In Figure 3, the state diagram of $G_{C}$ is presented. In the nonfaulty case, the diagram is reduced to that in [1,3,11].

### 2.4. The Model of the Drilling Machine with Faults

The model of the drilling machine, in the presence of faults, is expressed as $G_{D}=({\mathbb{Q}}_{D},E_{D},f_{D},{ℍ}_{D},x_{D,0},{\mathbb{Q}}_{D,m})$. The set of the states is ${\mathbb{Q}}_{D}=\{q_{D,1},q_{D,2},q_{D,3}\}$. The initial state is $x_{D,0}=q_{D,1}$. The set of the marked states is ${\mathbb{Q}}_{D,m}=\{q_{D,1}\}$. In Table 5, the states of the drilling machine are presented. The drilling machine is in faulty mode in cases of tool wear (see [26] and the references therein) or if the drilling tool is broken or one of the three linear actuators of the drilling machine is in faulty mode (see [11]), as well as if the drilling motor malfunctions, indicatively see [27,28]. The signals indicating the presence of such faults are derived through appropriate soft sensors that use the outputs of electric, speed and/or torque sensors, indicatively, see [26,27,28].

The alphabet is $E_{D}=\{e_{D,1},e_{D,2},e_{D,3},e_{D,4}\}$. In Table 6, the events of the drilling machine are presented. 

The 1st event is a command signal. The rest are appropriate observable signals. Thus, the set of the controllable events is $E_{D,c}=\{e_{D,1},e_{D,4}\}$ and the set of the uncontrollable events is $E_{D,uc}=\{e_{D,2},e_{D,3}\}$. The sets of the active events of $G_{D}$, are ${ℍ}_{D}(q_{D,1})=\{e_{D,1}\}$, ${ℍ}_{D}(q_{D,2})=\{e_{D,2},e_{D,3}\}$, ${ℍ}_{D}(q_{D,3})=\{e_{D,4}\}$. The values of transition function are $f_{D}(q_{D,1},e_{D,1})=q_{D,2}$, $f_{D}(q_{D,2},e_{D,2})=q_{D,1}$, $f_{D}(q_{D,2},e_{D,3})=q_{D,3}$, $f_{D}(q_{D,3},e_{D,4})=q_{D,1}$.

Note that $G_{D}$ is nonblocking, i.e., $\bar{L_{m}(G_{D})}=L(G_{D})$, where $L_{m}(G_{D})={\left(e_{D,1}(e_{D,2}+e_{D,3}e_{D,4})\right)}^{*}$. 

**Figure 4:**
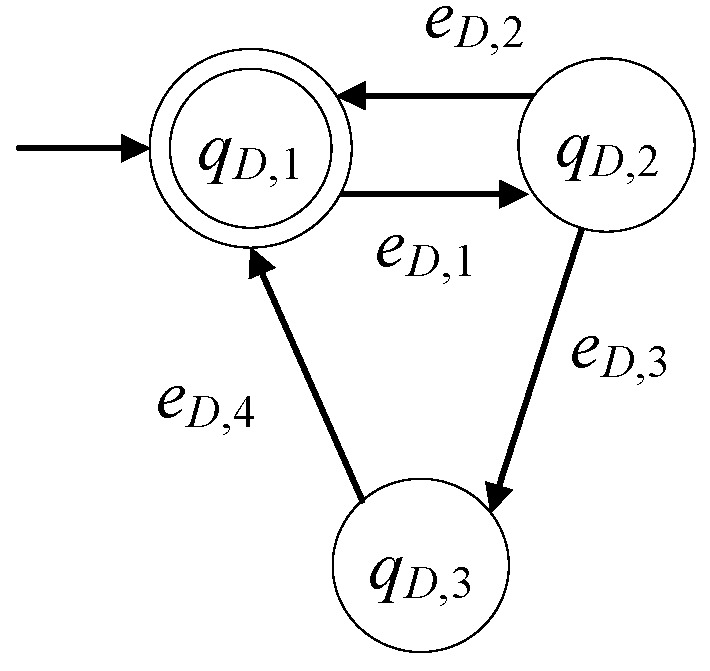
State diagram of $G_{D}$.

In Figure 4, the state diagram of the automaton of the drilling machine is presented. This diagram has first been presented in [1,3,11].

### 2.5. The Model of the Testing Device with Faults

Τhe model of the testing device, in the presence of faults, is developed to be of the six tuple form $G_{B}=({\mathbb{Q}}_{B},E_{B},f_{B},{ℍ}_{B},x_{B,0},{\mathbb{Q}}_{B,m})$. The set of the states is ${\mathbb{Q}}_{B}=\{q_{B,1},q_{B,2},q_{B,3}\}$. The initial state is $x_{B,0}=q_{B,1}$. The set of the marked states is ${\mathbb{Q}}_{B,m}=\{q_{B,1}\}$. In Table 7, the states of the testing device are presented. According to [11], the testing device consist of a linear actuator and a vacuum generator, as well as appropriate sensors. The testing device is in faulty mode for the reasons analogous to those presented for the C&T device, see also [24,25]. The repair signal can be produced in the same way to the previous subsystems and so is observable. 

The alphabet is $E_{B}=\{e_{B,1},e_{B,2},e_{B,3},e_{B,4},e_{B,5}\}$. In Table 8, the events of the testing device are presented.

The 1st and the 2nd event are command signals. The rest are observable signals. Thus, the set of the controllable events is $E_{B,c}=\{e_{B,1},e_{B,2},e_{B,5}\}$ and the set of the uncontrollable events is $E_{B,uc}=\{e_{B,3},e_{B,4}\}$. The sets of the active events of $G_{B}$ are

$${ℍ}_{B}(q_{B,1})=\{e_{B,1},e_{B,2}\},\;{ℍ}_{B}(q_{B,2})=\{e_{B,3},e_{B,4}\},\;{ℍ}_{B}(q_{B,3})=\{e_{B,5}\}.$$

The values of the transition function are

$$f_{B}(q_{B,1},e_{B,1})=q_{B,2},\;f_{B}(q_{B,1},e_{B,2})=q_{B,2},\;f_{B}(q_{B,2},e_{B,3})=q_{B,1},\;f_{B}(q_{B,2},e_{B,4})=q_{B,3},\;f_{B}(q_{B,3},e_{B,5})=q_{B,1}$$

The automaton $G_{B}$ is nonblocking, i.e., $\bar{L_{m}(G_{B})}=L(G_{B})$, where

$$L_{m}(G_{B})={\left(\left(e_{B,1}+e_{B,2})(e_{B,3}+e_{B,4}e_{B,5}\right)\right)}^{*}$$

**Figure 5:**
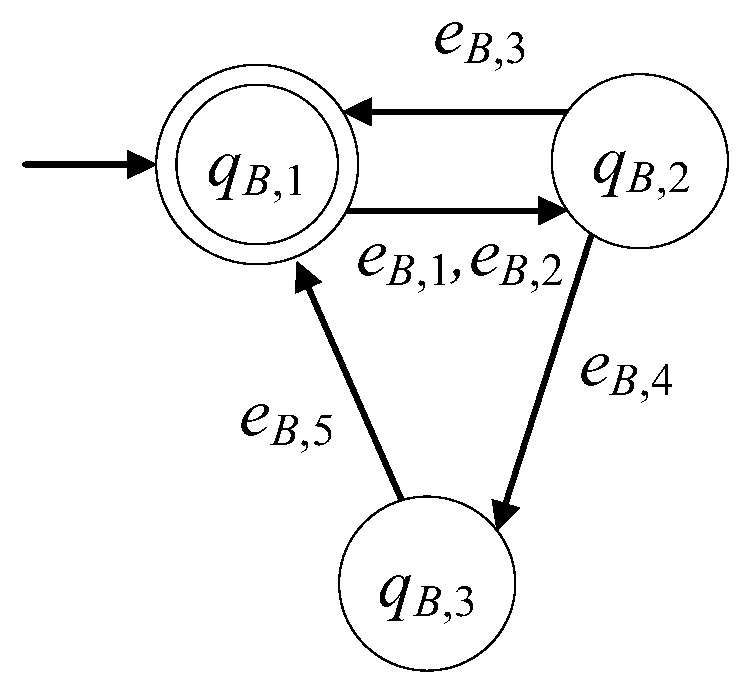
The state diagram of $G_{B}$.

In Figure 5, the state diagram of the automaton of the testing device is presented. In the nonfaulty case, the diagram is reduced to that in, see [1,3,11].

### 2.6. The Model of the Robotic Manipulator with Faults

The model of the robotic manipulator, in the presence of faults, is developed to be $G_{R}=({\mathbb{Q}}_{R},E_{R},f_{R},{ℍ}_{R},x_{R,0},{\mathbb{Q}}_{R,m})$. The set of the states is ${\mathbb{Q}}_{R}=\{q_{R,1},q_{R,2},q_{R,3},q_{R,4}\}$. The initial state is $x_{R,0}=q_{R,1}$. The set of the marked states is ${\mathbb{Q}}_{R,m}=\{q_{R,1}\}$. In Table 9, the states of the robotic manipulator are presented. The robotic manipulator can be in faulty mode for various reasons, indicatively see [29,30,31].

The alphabet is $E_{R}=\{e_{R,1},e_{R,2},e_{R,3},e_{R,4},e_{R,5}\}$. In Table 10, the events of the robotic manipulator are presented.

The 1st event is a command signal. The rest are observable signals. The controllable events set is $E_{R,c}=\{e_{R,1},e_{R,5}\}$ and the set of the uncontrollable events is $E_{R,uc}=\{e_{R,2},e_{R,3},e_{R,4}\}$. The sets of the active events of $G_{R}$ are

$${ℍ}_{R}(q_{R,1})=\{e_{R,1}\},\;{ℍ}_{R}(q_{R,2})=\{e_{R,2},e_{R,4}\},\;{ℍ}_{R}(q_{R,3})=\{e_{R,3},e_{R,4}\},\;{ℍ}_{R}(q_{R,4})=\{e_{R,5}\}$$

The values of the transition function of $G_{R}$ are

$$f_{R}(q_{R,1},e_{R,1})=q_{R,2},\;f_{R}(q_{R,2},e_{R,2})=q_{R,3},\;f_{R}(q_{R,2},e_{R,4})=q_{R,4},\;\;f_{R}(q_{R,3},e_{R,3})=q_{R,1},f_{R}(q_{R,3},e_{R,4})=q_{R,4},\;f_{R}(q_{R,4},e_{R,5})=q_{R,1}$$

$G_{R}$ is nonblocking automaton, i.e., $\bar{L_{m}(G_{R})}=L(G_{R})$, where

$$L_{m}(G_{R})={\left(e_{R,1}\left(e_{R,4}e_{R,5}+e_{R,2}(e_{R,3}+e_{R,4}e_{R,5})\right)\right)}^{*}$$

**Figure 6:**
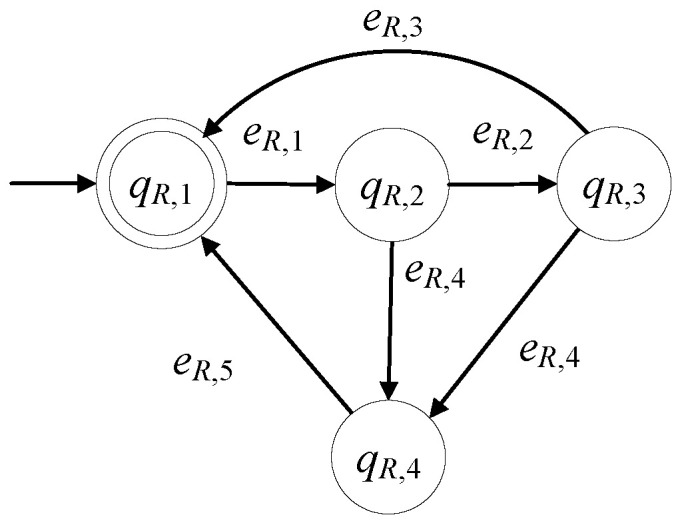
State diagram of $G_{R}$.

In Figure 6, the state diagram of $G_{R}$ is presented. In the nonfaulty case, the diagram is reduced to that in [1,3,11].

### 2.7. The Model of the Feeding Device with Faults

The model of the feeding device in the presence of faults is developed to be $G_{F}=({\mathbb{Q}}_{F},E_{F},f_{F},{ℍ}_{F},x_{F,0},{\mathbb{Q}}_{F,m})$. The set of the states is ${\mathbb{Q}}_{F}=\{q_{F,1},q_{F,2},q_{F,3},q_{F,4}\}$. The initial state is $x_{F,0}=q_{F,1}$. The set of the marked states is ${\mathbb{Q}}_{F,m}=\{q_{F,1}\}$. In Table 11 the description of the states of the feeding device are presented. According to [11] the feeding device consist of a linear actuator, a rotary actuator and appropriate sensors. The feeding device is in faulty mode for the same reasons to those presented for the C&T device, see [24,25,32]. The repair signal can be produced in the same way to the previous subsystems and so is observable.

The alphabet is $E_{F}=\{e_{F,1},e_{F,2},e_{F,3},e_{F,4},e_{F,5},e_{F,6}\}$. In Table 12, the events of the feeding device are presented.

The event $e_{F,1}$ is a command signal and the events $e_{F,2}$, $e_{F,3}$, $e_{F,4}$, $e_{F,5}$ and $e_{F,6}$ are observable signals. Thus, the set of the controllable events is $E_{F,c}=\{e_{F,1},e_{F,6}\}$ and the set of the uncontrollable events is $E_{F,uc}=\{e_{F,2},e_{F,3},e_{F,4},e_{F,5}\}$. The sets of the active events of $G_{F}$ are ${ℍ}_{F}(q_{F,1})=\{e_{F,1}\}$, ${ℍ}_{F}(q_{F,3})=\{e_{F,4},e_{F,5}\}$, ${ℍ}_{F}(q_{F,4})=\{e_{F,6}\}$. The values of the transition function are

$$f_{F}(q_{F,1},e_{F,1})=q_{F,2},\;f_{F}(q_{F,2},e_{F,2})=q_{F,1},\;f_{F}(q_{F,2},e_{F,3})=q_{F,3},\;f_{F}(q_{F,2},e_{F,5})=q_{F,4},\;f_{F}(q_{F,3},e_{F,4})=q_{F,1},\;f_{F}(q_{F,3},e_{F,5})=q_{F,4},\;\;f_{F}(q_{F,4},e_{F,6})=q_{F,1}$$

$G_{F}$ is a nonblocking automaton i.e., $\bar{L_{m}(G_{F})}=L(G_{F})$, where

$$L_{m}(G_{F})={\left(e_{F,1}\left(e_{F,2}+e_{F,5}e_{F,6}+e_{F,3}(e_{F,4}+e_{F,5}e_{F,6})\right)\right)}^{*}$$

**Figure 7:**
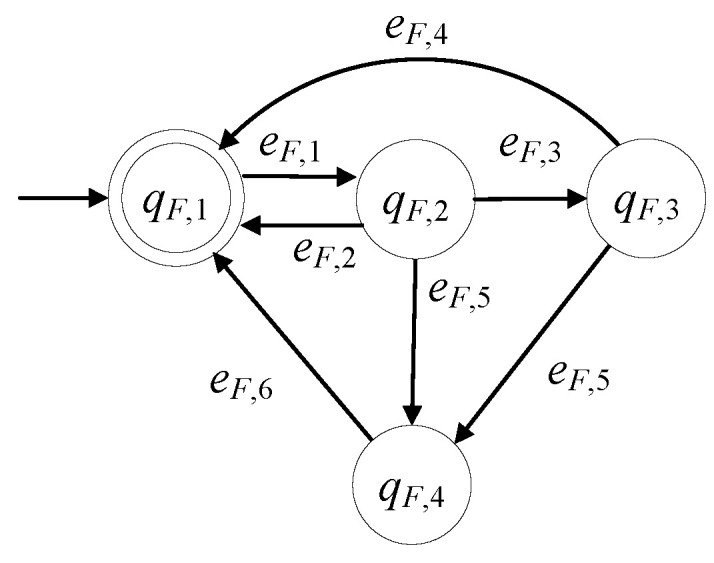
State diagram of $G_{F}$.

In Figure 7, the state diagram of $G_{F}$ is presented. In the nonfaulty case, the diagram is reduced to that in [1,3,11].

### 2.8. The Cell Model as a Shuffle

Since the event sets of the subsystems presented in Section 2 are disjoint sets, the model G of the manufacturing cell can be expressed as the shuffle [6] of the of the subsystems and can be expressed in the synchronous product form $G=G_{T}||G_{C}||G_{D}||G_{B}||G_{R}||G_{F}$. In [6,8], the definition and the properties of the synchronous product [6], or alternatively the parallel connection [8], are presented. The set of its states is $\mathbb{Q}={\mathbb{Q}}_{T}\times {\mathbb{Q}}_{C}\times {\mathbb{Q}}_{D}\times {\mathbb{Q}}_{B}\times {\mathbb{Q}}_{R}\times {\mathbb{Q}}_{F}$ and the states are of the form $q=(q_{T},q_{C},q_{D},q_{B},q_{R},q_{F})$. The alphabet of G is $E=E_{T}\cup E_{C}\cup E_{D}\cup E_{B}\cup E_{R}\cup E_{F}$. Clearly, all transitions of the subsystems are feasible. The active event sets of G satisfy the following property 

$ℍ\left(\left(q_{T},q_{C},q_{D},q_{B},q_{R},q_{F}\right)\right)={ℍ}_{T}(q_{T})\cup {ℍ}_{C}(q_{C})\cup {ℍ}_{D}(q_{D})\cup {ℍ}_{B}(q_{B})\cup {ℍ}_{R}(q_{R})\cup {ℍ}_{F}(q_{F})$ and the set of the marked states is ${\mathbb{Q}}_{m}=\{(q_{T,1},q_{C,1},q_{D,1},q_{B,1},q_{R,1},q_{F,1})\}$.

## 3. Desired Languages

In [11], a set of safety and functionality specifications has been presented. Here, the above specifications are enriched with requirements considering the possibility of the presence of the faults. Note that in the faulty case of the drilling machine the product is tested to be accepted or rejected. Here, except the drilling machine, after the detection of a fault to another subsystem and its repair, the process of the subsystem will reinitiate to complete the task with respect to the current product. 

In particular, the desired specifications, in the eventual presence of faults, are formulated, here, as follows:

1. When a fault takes place in the table or in the robotic manipulator, then the commands to leave from the idle state of the rest of the cell’s systems are deactivated until the fault’s repair.
2. The circular table is allowed to start rotating only if there is raw product in the appropriate position or a drilled piece in the drilling machine or a tested product in the testing device.
3. Table’s rotation and raw product transportation to the cell do not take place simultaneously.
4. Table’s rotation and drilling do not take place simultaneously.
5. Table’s rotation and testing do not take place simultaneously.
6. Table’s rotation and product retrieving, through the robotic manipulator, do not take place simultaneously.
7. The C&T device is not allowed to have two or more raw products in its output and the drilling machine is not allowed to start working without a product.
8. The drilling machine is not allowed to drill a product twice and the testing processes *A* and *B* of the testing device can begin only after the successful completion of the respective drilling process.
9. The robotic manipulator can retrieve a product only if there is a tested product in the respective position and the table’s rotation cannot initiate with a non-retrieved piece in the respective position.

The goal of the above rules is to protect the system from undesirable and/or malicious situations such as the ones described in the Section 1. Some possible malfunctions that may take place, are prevented by the following measures, being imposed by the nine specification rules,

- *Measure* 1: Unnecessary rotations of the table are avoided, as rule 2 does not allow table rotation without a product in one of the predefined positions of the table.
- *Measure* 2: Undesirable cooperation between the table and a device is prevented from rules 3–6 allowing table rotation only when the respective device is in idle mode.
- *Measure* 3: The case of overflow in the output of the C&T device and the case of drilling with no product in the respective position, are prevented from Rule 7.
- *Measure* 4: Product loss, through second drilling and/or testing of an unfinished product, is prevented from rule 8.
- *Measure* 5: Rule 9 prevents the loss of finished products.

The 1st specification can be decomposed to two prefixed closed regular languages. The first regular language is for the table of the system, while the second regular language is for the robotic manipulator

K11=((eC,1+eD,1+eB,1+eR,1+eF,1+eT,4)*eT,3(eT,3)*eT,4)*¯,K12=((eT,1+eC,1+eD,1+eB,1+eF,1+eR,5)*eR,4(eR,4)*eR,5)*¯

The 2nd specification is expressed by the following prefixed closed regular language:

K2=((eC,5+eD,2+eD,3+eB,3)(eC,5+eD,2+eD,3+eB,3)*eT,1)*¯

The 3rd specification is expressed by the following prefixed closed regular language:

K3=((eT,2+eT,4+eC,5+eC,6)*(eT,1+eC,4)(eT,2+eT,4+eC,5+eC,6))*¯.

The 4th specification is expressed by the following prefixed closed regular language:

K4=((eT,2+eT,4+eD,2+eD,4)*(eT,1+eD,1)(eT,2+eT,4+eD,2+eD,4))*¯.

The 5th specification is expressed by the following prefixed closed regular language:

K5=((eT,2+eT,4+eB,3+eB,5)*(eT,1+eB,1+eB,2)(eT,2+eT,4+eB,3+eB,5))*¯.

The 6th specification is expressed by the following prefixed closed regular language:

K6=((eT,2+eT,4+eR,2+eR,5)*(eT,1+eR,1)(eT,2+eT,4+eR,2+eR,5))*¯

The 7th specification is expressed by the following prefixed closed regular language:

K7=((eT,1+eC,4)*eC,5(eC,5)*eT,1(eC,5)*(eC,4(eC,5)*+ε)*eD,1)*¯

The 8th specification can be analyzed to the following two prefixed closed regular languages:

K81=((eD,1)*eD,2(eD,2)*eB,1)*¯,K82=((eD,1)*eD,3(eD,3)*eB,2)*¯.

The 9th specification is expressed by the following prefixed closed regular language

K9=((eT,1)*eB,3(eB,3)*eT,1(eB,3)*eR,1)*¯

Using the same indices, the alphabets of the above prefixed closed regular languages are

E1S,1={eC,1,eD,1,eB,1,eR,1,eF,1,eT,3,eT,4}, E1S,2={eT,1,eC,1,eD,1,eB,1,eF,1,eR,4,eR,5},E2S={eT,1,eC,5,eD,2,eD,3,eB,3},E3S={eT,1,eT,2,eT,4,eC,4,eC,5,eC,6},E4S={eT,1,eT,2,eT,4,eD,1,eD,2,eD,4}, E5S={eT,1,eT,2,eT,4,eB,1,eB,2,eB,3,eB,5},E6S={eT,1,eT,2,eT,4,eR,1,eR,2,eR,5}, E7S={eT,1,eC,4,eC,5,eD,1},E8S,1={eD,1,eD,2,eB,1}, E8S,2={eD,1,eD,3,eB,2}, E9S={eT,1,eB,3,eR,1}

To satisfy the specifications 1–9, the automaton G will be controlled by appropriate supervisors. To this end, similarly to [18,33] and because the specifications are expressed by prefixed closed languages, the performance of the resulting controlled automaton is proposed to be described by the following 11 desired languages

(1) K1D,1=P11−1(K11¯)∩Lm(G)=P11−1(K11)∩Lm(G)

(2) K1D,2=P12−1(K12¯)∩Lm(G)=P12−1(K12)∩Lm(G)

(3) K2D=P2−1(K2¯)∩Lm(G)=P2−1(K2)∩Lm(G)

(4) K3D=P3−1(K3¯)∩Lm(G)=P3−1(K3)∩Lm(G)

(5) K4D=P4−1(K4¯)∩Lm(G)=P4−1(K4)∩Lm(G)

(6) K5D=P5−1(K5¯)∩Lm(G)=P5−1(K5)∩Lm(G)

(7) K6D=P6−1(K6¯)∩Lm(G)=P6−1(K6)∩Lm(G)

(8) K7D=P7−1(K7¯)∩Lm(G)=P7−1(K7)∩Lm(G)

(9) K8D,1=P81−1(K81¯)∩Lm(G)=P81−1(K81)∩Lm(G)

(10) K8D,2=P82−1(K82¯)∩Lm(G)=P82−1(K82)∩Lm(G)

(11) K9D=P9−1(K9¯)∩Lm(G)=P9−1(K9)∩Lm(G)

where P11 and P12 denote the projections of $E^{*}$ to E1S,1* and E1S,2*, respectively. P81 and P82 denote the projections of $E^{*}$ to E8S,1* and E8S,2*, respectively. P2 till P7 denote the projections of $E^{*}$ to E2S* till E7S*, respectively. P9 denotes the projection of $E^{*}$ to E9S*.

## 4. Supervisors

### 4.1. Notation and Properties of Supervisory Design

In order to control an automaton, let G, a finite deterministic automaton, called supervisor and denoted by $S=({\mathbb{Q}}_{S},E_{S},f_{S},{ℍ}_{S},x_{S,0},{\mathbb{Q}}_{S,m})$, will be used. The closed and the marked behavior of the controlled automaton by the aforementioned supervisor are equal to the closed and the marked behavior of the synchronous product [6] (or parallel composition [8]) of S and G, denoted by S||G. The complexity of S (indicatively see [13,34]) is the triad including the number of the states, the number of the events and the number of the transitions of S.

The control action of S to G is physical realizable (PR) (see [35]) if the transitions of G due to its uncontrollable events are not disactivated by S||G. The performance of the controlled automaton is nonblocking if S||G is a nonblocking automaton, see [6,8]. Here, the case of multiple supervisors in a modular scheme will be used. For more details about the modular supervisory design, see [6,8] and the extensions developed in [18,19,20,21,22,23,24,25,26,27,28,29,30,31,32,33].

### 4.2. A Two-State Supervisor form Realizing the First Six and The 8th Specifications

The automaton $S_{1}=({\mathbb{Q}}_{S,1},E_{S,1},f_{S,1},{ℍ}_{S,1},x_{S,1,0},{\mathbb{Q}}_{S,1,m})$ denotes a class of supervisors. The cardinality of the set of the states of $S_{1}$ is equal to 2, i.e., ${\mathbb{Q}}_{S,1}=\{q_{S,1,1},q_{S,1,2}\}$. The class of supervisors depends upon four regular expressions, denoted by c11, c12, c13 and c14. For the definition and properties of regular expressions, see [6,8]. Their alphabets are denoted by E1c,1, E1c,2, E1c,3 and E1c,4, respectively. The alphabet of $S_{1}$ is ES,1=E1c,1∪E1c,2∪E1c,3∪E1c,4. The initial state of $S_{1}$ is $x_{S,1,0}=q_{S,1,1}$. It is considered that all states are marked, i.e., ${\mathbb{Q}}_{S,1,m}={\mathbb{Q}}_{S,1}$. The active event sets of $S_{1}$ are ℍS,1(qS,1,1)=E1c,1∪E1c,2 and ℍS,1(qS,1,2)=E1c,3∪E1c,4. The values of the transition function of $S_{1}$ are

fS,1(qS,1,1,e)=qS,1,1, ∀e∈E1c,1, fS,1(qS,1,1,e)=qS,1,2, ∀e∈E1c,2,fS,1(qS,1,2,e)=qS,1,2, ∀e∈E1c,3, fS,1(qS,1,2,e)=qS,1,1, ∀e∈E1c,4

**Figure 8:**
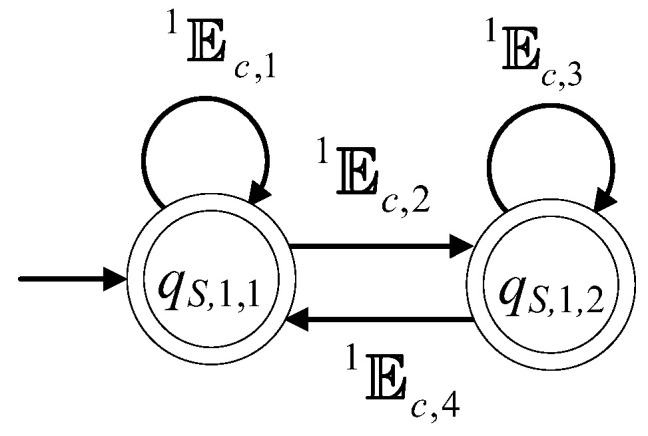
State diagram of $S_{1}$.

The complexity triad of $S_{1}$ is $\left(2,\;|E_{S,1}|,|E_{S,1}|\right)$, where |•| denotes the cardinality of the argument set. The state diagram of $S_{1}$ is depicted in Figure 8. 

$S_{1}$ will be used for the realization of seven automata, where their closed and the marked behaviors will be equal to the prefixed closed regular languages K11, K12, K2, K3, K4,K5 and K6, respectively. In Table 13, the supervisor’s symbol derived, using $S_{1}$, the respective languages and their complexity triad, are presented. According to Table 13, the alphabets of the regular expressions are uniquely determined. Indicatively, for S11,1 it holds that E1c,1={eC,1,eD,1,eB,1,eR,1,eF,1}, E1c,2=E1c,3={eT,3} and E1c,4={eT,4}.

### 4.3. A Three-State Supervisor Realizing the 9th Specification

The supervisor automaton, realizing K9, is of the form $S_{2}=({\mathbb{Q}}_{S,2},E_{S,2},f_{S,2},{ℍ}_{S,2},x_{S,2,0},{\mathbb{Q}}_{S,2,m})$. The set of the states of $S_{2}$ is ${\mathbb{Q}}_{S,2}=\{q_{S,2,1},q_{S,2,2},q_{S,2,3}\}$ and $|{\mathbb{Q}}_{S,2}|=3$. The alphabet of $S_{2}$ is ES,2=E9S. Its initial state is denoted by $x_{S,2,0}=q_{S,2,1}$. All states of $S_{2}$ are marked, i.e., ${\mathbb{Q}}_{S,2,m}={\mathbb{Q}}_{S,2}$. The sets of the active events, per state of $S_{2}$, are ${ℍ}_{S,2}(q_{S,2,1})=\{e_{T,1},e_{B,3}\}$, ${ℍ}_{S,2}(q_{S,2,2})=\{e_{T,1},e_{B,3}\}$ and ${ℍ}_{S,2}(q_{S,2,3})=\{e_{B,3},e_{R,1}\}$. The values of the transition function of $S_{2}$ are

$$f_{S,2}(q_{S,2,1},e_{T,1})=q_{S,2,1},\;f_{S,2}(q_{S,2,1},e_{B,3})=q_{S,2,2},\;f_{S,2}(q_{S,2,2},e_{B,3})=q_{S,2,2},\;f_{S,2}(q_{S,2,2},e_{T,1})=q_{S,2,3},\;f_{S,2}(q_{S,2,3},e_{B,3})=q_{S,2,3},\;f_{S,2}(q_{S,2,3},e_{R,1})=q_{S,2,1}$$

**Figure 9:**
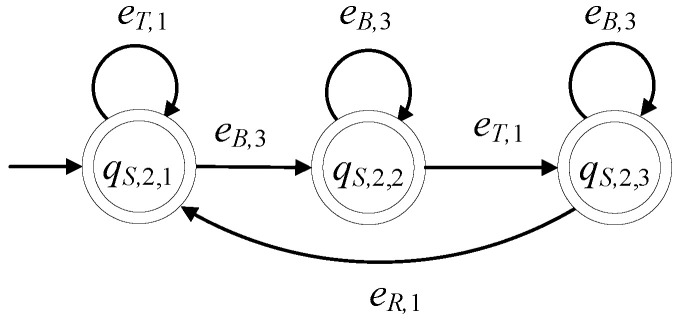
State diagram of $S_{2}$.

The complexity triad of $S_{2}$ is (3, 3, 6). Its state diagram is presented in Figure 9. 

### 4.4. A Four-State Supervisor Realizing the 7th Specification

The supervisor automaton, realizing K7, is of the form $S_{3}=({\mathbb{Q}}_{S,3},E_{S,3},f_{S,3},{ℍ}_{S,3},x_{S,3,0},{\mathbb{Q}}_{S,3,m})$. The set of the states of $S_{3}$ is ${\mathbb{Q}}_{S,3}=\{q_{S,3,1},q_{S,3,2},q_{S,3,3},q_{S,3,4}\}$ and $|{\mathbb{Q}}_{S,3}|=4$. The alphabet of $S_{3}$ is ES,3=E7S. Its initial state is denoted by $x_{S,3,0}=q_{S,3,1}$. All states of $S_{3}$ are marked, i.e., ${\mathbb{Q}}_{S,3,m}={\mathbb{Q}}_{S,3}$. The sets of the active events, per state of $S_{3}$, are

$${ℍ}_{S,3}(q_{S,3,1})=\{e_{T,1},e_{C,4},e_{C,5}\},\;{ℍ}_{S,3}(q_{S,3,2})=\{e_{T,1},e_{C,5}\},\;{ℍ}_{S,3}(q_{S,3,3})=\{e_{D,1},e_{C,4}\}\;\mathrm{and}\;{ℍ}_{S,3}(q_{S,3,4})=\{e_{D,1},e_{C,5}\}$$

The values of the transition function of $S_{3}$ are

$$f_{S,3}(q_{S,3,1},e_{T,1})=q_{S,3,1},\;f_{S,3}(q_{S,3,1},e_{C,4})=q_{S,3,1},\;f_{S,3}(q_{S,3,1},e_{C,5})=q_{S,3,2},\;\;f_{S,3}(q_{S,3,2},e_{C,5})=q_{S,3,2},\;f_{S,3}(q_{S,3,2},e_{T,1})=q_{S,3,3},\;f_{S,3}(q_{S,3,3},e_{C,5})=q_{S,3,3},\;\;f_{S,3}(q_{S,3,3},e_{D,1})=q_{S,3,1},\;f_{S,3}(q_{S,3,3},e_{C,4})=q_{S,3,4},\;f_{S,3}(q_{S,3,4},e_{C,5})=q_{S,3,4},\;\;f_{S,3}(q_{S,3,4},e_{D,1})=q_{S,3,1}$$

**Figure 10:**
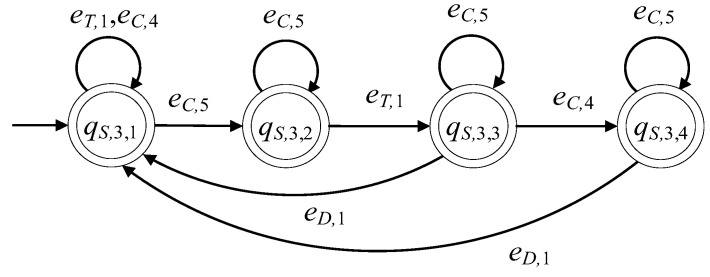
State diagram of $S_{3}$.

The complexity triad of $S_{3}$ is (4, 4, 10). Its state diagram is presented in Figure 10. 

## 5. The Performance of the Controlled Automaton

The supervisors proposed in Section 4, are interconnected to the automaton G of the manufacturing cell through the following multi argument synchronous product

(12) Gc=S11,1||S11,2||S21||S31||S41||S51||S61||S81,1||S81,2||S2||S3||G.

Automaton $G_{c}$ is the controlled automaton. In this section, the performance of the controlled automaton $G_{c}$ will be investigated. It will be proven that $G_{c}$ satisfies the desired specifications 1–9, presented in Section 3. To this end and using the properties of the multi argument synchronous product (see [9,10]) and the property that the closed and the marked behaviors of the supervisors, are equal to the prefixed closed regular languages K11, K12, K2, K3, K4, K5, K6, K7, K81, and K9, respectively, the closed behavior and the marked behavior of $G_{c}$ will first be computed

(13) L(Gc)=L(G)∩[∩λ=27Pλ−1(Kλ¯)]∩P11−1(K11¯)∩∩P12−1(K12¯)∩P81−1(K81¯)∩P82−1(K82¯)∩P9−1(K9¯)

(14) Lm(Gc)=Lm(G)∩[∩λ=27Pλ−1(Kλ)]∩P11−1(K11)∩∩P12−1(K12)∩P81−1(K81)∩P82−1(K82)∩P9−1(K9)==K1D,1∩K2D,1∩[∩λ=27KλD]∩K8D,1∩K8D,2∩K9D

From (14), it is observed that the performance of the controlled automaton $G_{c}$, regarding its marked behavior, is satisfactory. Regarding the closed behavior of $G_{c}$, it is mentioned that in order to be satisfactory it is necessary and sufficient that $G_{c}$ is nonblocking, i.e., $\bar{L_{m}(G_{c})}=L(G_{c})$. This property will be proven in Proposition 2. 

**Figure 11:**
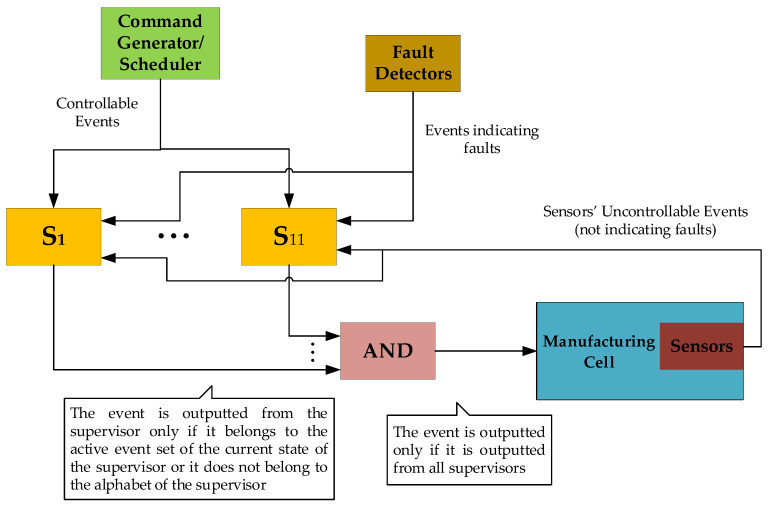
Operational flow chart of the supervisory control scheme.

In Figure 11, the operational flow of the present modular supervisory scheme is presented. The symbols $S_{1}$ to $S_{11}$ represent the eleven supervisors of the present control scheme (see Section 4). All commands (controllable events) are generated by the Generator/Scheduler and inputted to the eleven supervisors. All sensors’ signals (uncontrollable events) are produced by the sensors and inputted to the eleven supervisors. The indications of faults (uncontrollable events) are produced by Fault Detectors. The outputs of all supervisors are connected to an “AND” block. An event is outputted by this block only if it is outputted by all eleven supervisors. The above algorithm is the main idea of modular supervising control.

Before examining the closed behavior of the controlled automaton, it is necessary to examine the physical realizability (PR) of the synchronous product in Relation (12). The physical realizability (see [35,36]) is translated into the condition that the transitions of G, activated by uncontrollable events, must not obstructed by the twelve supervisors.

**Proposition 1**:  *The synchronous product of the designed supervisor scheme is PR, with respect to* G *, through (12).*

**Proof of Proposition 1**:  It holds that ℍ1S,1,1(q1S,1,1)∩E1S,1,uc=E1S,1,uc, where E1S,1,uc=E1S,1∩Euc, and E1S,1⊂E. Using Corollary 1 in [35], it is concluded that S11,1 is PR with respect to G, through S11,1||G. The alphabet and the set of the uncontrollable events of S11,1||G are equal to the respective sets of G. Also, it holds that ℍ1S,2(q1S,1,2)∩E1S,2,uc=E1S,2,uc and E1S,2⊂E, where E1S,2,uc=E1S,2∩Euc. Hence, using Corollary 1 of [32], it is concluded that S11,2 is PR with respect to S11,1||G, through S11,2||S11,1||G. It holds that ℍiS,1(qiS,1,1)∩EiS,uc=EiS,uc, where EiS,uc=EiS∩Euc and i∈{2,…,6}, as well as that EiS⊂E. The alphabet and the set of the uncontrollable events of (||λ=2i−1Sλ1)||S11,2||S11,1||G are equal to the respective sets of G. Hence, using Corollary 1 of [32], it is concluded that Si1 is PR, with respect to (||λ=2i−1Sλ1)||S11,2||S11,1||G, through Si1||(||λ=2i−1Sλ1)||S11,2||S11,1||G. The alphabet and the set of the uncontrollable events of (||λ=26Sλ1)||S11,2||S11,1||G are equal to the respective sets of G. It holds that E8S,1⊂E, ℍ8S,1,1(q8S,1,1)∩E8S,1,uc=E8S,1,uc, where E8S,1,uc=E8S,1∩Euc. Hence, using Corollary 1 of [32], it is concluded that S81,1 is PR, with respect to (||λ=26Sλ1)||S11,2||S11,1||G, through S18||(||λ=26(Sλ1))||S11,2||S11,1||G. The alphabet and the set of the uncontrollable events of S18||(||λ=26(Sλ1))||S11,2||S11,1||G are equal to the respective sets of **G**. Clearly, it holds that E8S,2⊂E, ℍ8S,1,2(q8S,1,2)∩E8S,2,uc=E8S,2,uc, where E8S,2,uc=E8S,2∩Euc. Thus, using Corollary 1 of [35], it is concluded that S81,2 is PR, with respect to S81||(||λ=26(Sλ1))||S11,2||S11,1||G, through S81,2||S81,1||(||λ=26Sλ1)||S11,2||S11,1||G. The alphabet and the set of the uncontrollable events of S81,2||S81,1||(||λ=26Sλ1)||S11,2||S11,1||G are equal to the respective sets of G. Also, it holds that $E_{S,2}\subset E$, ${ℍ}_{S,2}(q_{S,2})\cap E_{S,2,uc}=E_{S,2,uc}$, where $E_{S,2,uc}=E_{S,2}\cap E_{uc}$. Thus, using Corollary 1 of [32], it is concluded that $S_{2}$ is PR with respect to

S81,2||S81,1||(||λ=26Sλ1)||S11,2||S11,1||G,

through S2||S81,2||S81,1||(||λ=26Sλ1)||S11,2||S11,1||G. Finally, the alphabet and the set of the uncontrollable events of S2||S81,2||S81,1||(||λ=26Sλ1)||S11,2||S11,1||G are equal to the respective sets of G. Also, it holds that $E_{S,3}\subset E$, ${ℍ}_{S,3}(q_{S,3})\cap E_{S,3,uc}=E_{S,3,uc}$, where $E_{S,3,uc}=E_{S,3}\cap E_{uc}$. Hence, using Corollary 1 of [32], it is concluded that $S_{3}$ is PR, with respect to

S2||S81,2||S81,1||(||λ=26Sλ1)||S11,2||S11,1||G,

through S3||S2||S81,2||S81,1||(||λ=26Sλ1)||S11,2||S11,1||G. □

**Proposition 2**:  *The controlled automaton*$G_{c}$*is a nonblocking automaton*.

**Proof of Proposition 2**:  Next, the six automata of the corresponding subsystems under the influence of the twelve supervisors will be examined regarding the nonblocking property. It is important to mention that all supervisors are physical realizable regarding G, i.e., all desired languages are controllable regarding G. In what follows, it will be investigated if there are direct (single step) or indirect (more than one steps) transitions from the non-marked states of $G_{c}$ to marked states of $G_{c}$. Since all states of the supervisors are marked, all non-marked states of $G_{c}$ include as a component at least one non-marked state of the subsystems of G. To this end, for all non-marked states of each subsystem of G, it will be investigated if there is a direct or indirect transition, not obstructed by the supervisor and the rest subsystems, that moves the subsystem to a marked state. Since G is the shuffle of its subsystems, this transition will not be related to any transition of the rest subsystems of G. Thus, the aforementioned investigation will form a procedure, where upon checking one subsystem of G, after the appropriate transition, the number of the non-marked state components of a non-marked state of $G_{c}$ will be decreased by one. So, at the end of the procedure $G_{c}$ will arrive at a marked state and the proof will be completed.

Starting the investigation with $G_{T}$, it is observed that it has two non-marked states, namely, the states $q_{T,2}$ and $q_{T,3}$. Regarding $q_{T,2}$, it is recalled that ${ℍ}_{T}(q_{T,2})=E_{T,uc}=\{e_{T,2},e_{T,3}\}$, $f_{T}(q_{T,2},e_{T,2})=q_{T,1}$. Since all supervisors are physical realizable, with respect to G, it is observed that they are also PR, with respect to $G_{T}$. Using this observation and the property that G is a shuffle, it is concluded that the transition from $q_{T,2}$ to the marked state $q_{T,1}$, is always feasible using the uncontrollable event $e_{T,2}$. Regarding $q_{T,3}$, it holds that ${ℍ}_{T}(q_{T,3})=\{e_{T,4}\}$ and $f_{T}(q_{T,3},e_{T,4})=q_{T,1}$. It is observed that only the supervisors S11,1,S31,S41,S51 and S61 have the event $e_{T,4}$ in their alphabet and that the event $e_{T,4}$ is in the active event sets of all supervisors’ states. Hence, the transition from $q_{T,3}$ to the marked state $q_{T,1}$, using $e_{T,4}$ is not obstructed by the afore mentioned supervisors as well as the rest supervisors as they do not include $e_{T,4}$ in their alphabets. Also, recall that this transition is not obstructed by the rest subsystems, i.e., $G_{C}$, $G_{D}$, $G_{B}$, $G_{R}$ and $G_{F}$, as their alphabets are disjoint sets with respect to $E_{T}$. Hence, the transition from any state of $G_{c}$, including as a component a non-marked state of $G_{T}$, to a state of $G_{c}$, where the non-marked state of $G_{T}$ has been substituted by a marked state of $G_{T}$, is always feasible.

The investigation will continue with $G_{C}$. The automaton $G_{C}$ has four non-marked states, namely the states $q_{C,2}$, $q_{C,3}$, $q_{C,4}$ and $q_{C,5}$. The repair event $e_{C,7}$ does not belong to any supervisor alphabet. Thus, the transition from the non-marked state $q_{C,5}$ to the marked state $q_{C,1}$ is always active, as $f_{C}(q_{C,5},e_{C,7})=q_{C,1}$. Regarding $q_{C,2}$ and $q_{C,4}$, it holds that ${ℍ}_{C}(q_{C,2})=\{e_{C,3}\}$, $f_{C}(q_{C,2},e_{C,3})=q_{C,1}$, ${ℍ}_{C}(q_{C,4})=\{e_{C,5}\}$, $f_{C}(q_{C,4},e_{C,5})=q_{C,1}$; $e_{C,3},e_{C,5}\in E_{C,uc}$. Hence, taking into account the physical realizability of the proposed supervisory scheme regarding G and the property that G is a shuffle, the transition from $q_{C,2}$ and $q_{C,4}$ to the marked state $q_{C,1}$ is always feasible using uncontrollable events. Finally, regarding $q_{C,3}$ it holds that ${ℍ}_{C}(q_{C,3})=\{e_{C,4},e_{C,6}\}$ and $f_{C}(q_{C,3},e_{C,4})=q_{C,4}$. It is observed that only the supervisors S31 and S71 have the event $e_{C,4}$ in their alphabet. Regarding S31, it is observed that in the first state the event $e_{C,4}$ is active. In the second state, the event $e_{C,4}$ is not active but there is always an active transition to the first state using an uncontrollable event. Thus, S31 does not obstruct the transition from $q_{C,3}$ to $q_{C,4}$. Regarding S71, it is observed that in the first and third state, the event $e_{C,4}$ is active. In the second and fourth state the event $e_{C,4}$ is not active but there is always an active transition to the third and first state using uncontrollable events. Thus, S71 does not obstruct the transition from $q_{C,3}$ to $q_{C,4}$. Also, the rest supervisors, namely all supervisors except S31 and S71 do not obstruct this transition, as they do not include $e_{C,4}$ in their alphabets. Regarding the automata $G_{T}$, $G_{D}$, $G_{B}$, $G_{R}$ and $G_{F}$, their alphabets are disjoint sets with respect to $E_{C}$. Hence, the transition from any state of $G_{c}$ including as a component a non-marked state of $G_{C}$, to a state of $G_{c}$ where the non-marked state of $G_{C}$ has been substituted by a marked state of $G_{C}$, is always feasible.

The automaton $G_{D}$ has two non-marked states, namely the states $q_{D,2}$ and $q_{D,3}$. Regarding $q_{D,2}$, it is recalled that ${ℍ}_{D}(q_{D,2})=E_{D,uc}$, $f_{D}(q_{D,2},e_{D,2})=q_{D,1}$. Since all supervisors are physical realizable with respect to $G_{D}$, the transition from $q_{D,2}$ to the marked state $q_{D,1}$, using the uncontrollable event $q_{D,2}$ is always feasible. Finally, regarding, $q_{D,3}$ it holds that ${ℍ}_{D}(q_{D,3})=\{e_{D,4}\}$ and $f_{D}(q_{D,3},e_{D,4})=q_{D,1}$. It is observed that only the supervisor S41 has the event $e_{D,4}$ in its alphabet. The event $e_{D,4}$ is active in all states of S41. Hence, the transition from $q_{D,3}$ to the marked state $q_{D,1}$, using $e_{D,4}$, is not obstructed by S41. Obviously, it is also not obstructed by the rest of the subsystems $G_{T}$, $G_{C}$, $G_{B}$, $G_{R}$ and $G_{F}$, as their alphabets are disjoint sets with respect to $E_{D}$. Hence, the transition from any state of $G_{c}$, including as a component a non-marked state of $G_{D}$, to a state of $G_{c}$, where the non-marked state of $G_{D}$ has been substituted by a marked state of $G_{D}$, is always feasible.

The automaton $G_{B}$ has also two non-marked states, namely the states $q_{B,2}$ and $q_{B,3}$. Regarding $q_{B,2}$, it is recalled that ${ℍ}_{B}(q_{B,2})=E_{B,uc}$, $f_{B}(q_{B,2},e_{B,3})=q_{B,1}$. Since all supervisors are physical realizable, with respect to $G_{B}$, the transition from $q_{B,2}$ to the marked state $q_{B,1}$ is always feasible using the uncontrollable event $e_{B,3}$. Finally, regarding $q_{B,3}$, it holds that ${ℍ}_{B}(q_{B,3})=\{e_{B,5}\}$ and $f_{B}(q_{B,3},e_{B,5})=q_{B,1}$. It is observed that only the supervisor S51 has the event $e_{B,5}$ in its alphabet. The event $e_{B,5}$ is active for all states of S51. Hence, the transition from $q_{B,3}$ to the marked state $q_{B,1}$, using $e_{B,5}$, is not obstructed by S51. Obviously, this transition is not obstructed by the rest of the subsystems $G_{T}$, $G_{C}$, $G_{D}$, $G_{R}$ and $G_{F}$, as their alphabets are disjoint sets with respect to $E_{B}$. Hence, the transition from any state of $G_{c}$, including as a component a non-marked state of $G_{B}$, to a state of $G_{c}$, where the non-marked state of $G_{B}$ has been substituted by a marked state of $G_{B}$, is always feasible.

The automaton $G_{R}$ has three non-marked states, namely the states $q_{R,2}$, $q_{R,3}$ and $q_{R,4}$. Regarding $q_{R,2}$ and $q_{R,3}$, it holds that ${ℍ}_{R}(q_{R,2})=\{e_{R,2},e_{R,4}\}$, $f_{R}(q_{R,2},e_{R,2})=q_{R,3}$, ${ℍ}_{R}(q_{R,3})=\{e_{R,3},e_{R,4}\}$, $f_{R}(q_{R,3},e_{R,3})=q_{R,1}$, where $e_{R,2},e_{R,3}\in E_{R,uc}$. Hence, taking into account the physical realizability of the proposed supervisory scheme, with respect to G, and the property that G is a shuffle, it is observed that the transitions from $q_{R,2}$ and $q_{R,3}$ to the marked state $q_{R,1}$ are always feasible using uncontrollable events. Finally, regarding $q_{R,4}$, it holds that ${ℍ}_{R}(q_{R,4})=\{e_{R,5}\}$ and $f_{R}(q_{R,4},e_{R,5})=q_{R,1}$. It is observed that only the supervisors S11,2 and S61 have the event $e_{R,5}$ in their alphabet. The event $e_{R,5}$ belongs the active event sets of all states of these two supervisors. Hence, the transition from $q_{R,4}$ to the marked state $q_{R,1}$, using $e_{R,5}$, is not obstructed by S11,2 and S61 as well as the rest supervisors as they do not include $e_{R,5}$ in their alphabets. Also, this transition is not obstructed by the rest of the subsystems, namely $G_{T}$, $G_{C}$, $G_{D}$, $G_{B}$ and $G_{F}$, as their alphabets are disjoint sets with respect to $E_{R}$. Hence, the transition from any state of $G_{c}$, including as a component a non-marked state of $G_{R}$, to a state of $G_{c}$, where the non-marked state of $G_{R}$ has been substituted by a marked state of $G_{R}$, is always feasible. Thus, the automaton $G_{R,c}$ is a nonblocking automaton. □

**Remark 1**:  *The nonblocking property of $G_{c}$ is guaranteed regardless the consideration of faults in the subsystems, in the sense that the transitions from states of $G_{c}$, being non-faulty and non-marked, to marked states of $G_{c}$ are feasible without necessarily passing through faulty states.*

**Remark 2**:  *The complexity of the proposed modular supervisor scheme is (25, 57, 101).*

**Remark 3**:  *The design of the first six supervisors and the 8-th supervisor are realized by a common parametric function block. The above characteristic contributes to the controller implementation facilitating the respective programming.*

## 6. The Case without Faults

In the case where there are no faults in the system, the models of the subsystems automata presented in Section 2, are reduced to appropriate sub-automata, denoted by ${\tilde{G}}_{T}$, ${\tilde{G}}_{C}$, ${\tilde{G}}_{D}$, ${\tilde{G}}_{B}$, ${\tilde{G}}_{R}$ and ${\tilde{G}}_{F}$. The above automata are derived by removing the faulty states, the events of the faults and the fault repair events as well as any transition related to the above states and events of the respective automata presented in Section 2. Also, in the no faults case, the desired languages presented in Section 3, are reduced to a set of new languages, denoted by K˜11, K˜12, K˜2, K˜3, K˜4, K˜5, K˜6, K˜7, K˜81, K˜82 and K˜9. The above languages are derived by the respective languages of the faulty case, upon substituting the events of faults and the fault repair events with the empty word ε. Finally, the supervisors realizing these new languages, S˜11,1, S˜11,2, S˜21, S˜31, S˜41, S˜51, S˜61, S˜81,1, S˜81,2, ${\tilde{S}}_{2}$ and ${\tilde{S}}_{3}$. The above supervisors are derived by the respective supervisors of the faulty case, upon removing the events of the faults and the fault repair events as well as the transitions, related only to these events, and finally upon removing the non-accessible states. It is important to mention that the supervisors S˜11,1 and S˜11,2, handle the faults of the table and the faults of the robotic manipulator, will now be single state automata with self-transitions triggered by all events in the alphabet of each supervisor. Hence, in the case without faults, the above two supervisors do not contribute to the performance of the control system and so they can be neglected. 

In concluding, in the case without faults, the proposed here supervisory scheme is still effective.

## 7. Supervisor Implementation

An interesting issue, on the implementation of a supervisor control scheme, is the transition from event-based supervisors, built in the form of automata models, to standard signal-based PLC’s operation, see [1,3,11]. To demonstrate the ease implementation of the proposed, here, supervisory control scheme, in real time industrial controllers such as PLCs, PACs etc., the present supervisors are implemented in the international standard IEC 61131–3 (2013). Industry 4.0 trends for real time industrial controller implementation, can be found in [33] and the references therein. Also, details regarding programming for the implementation of supervisor automata can be found in [1,5] and the refences therein. 

**Figure 12:**
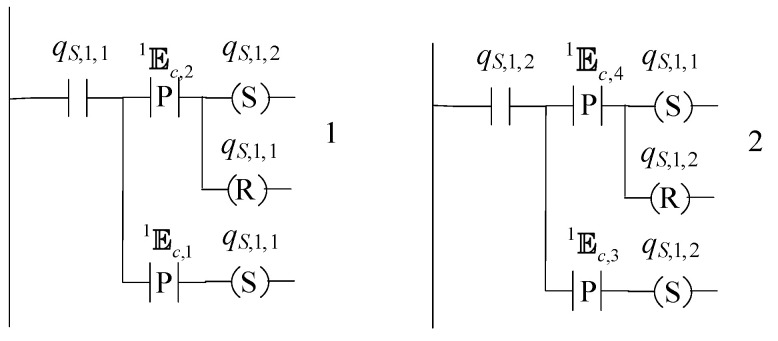
State diagram of $S_{1}$.

**Figure 13:**
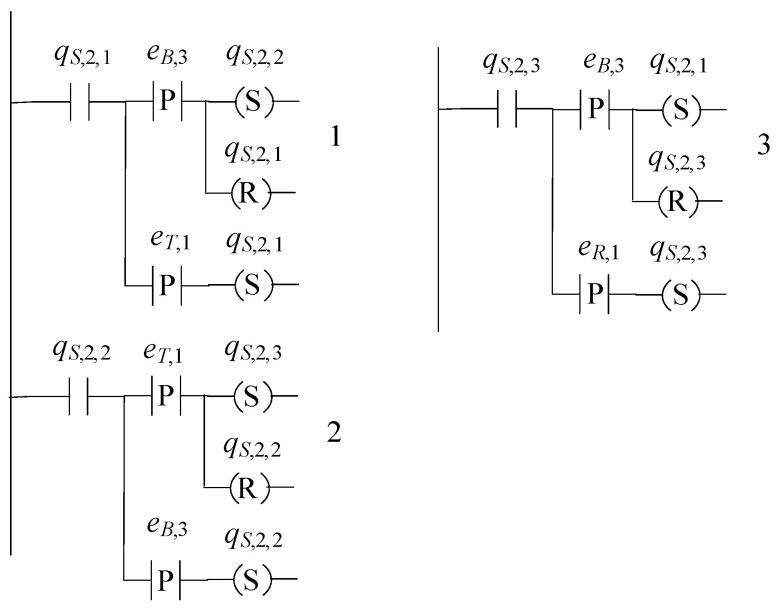
State diagram of $S_{2}$.

**Figure 14:**
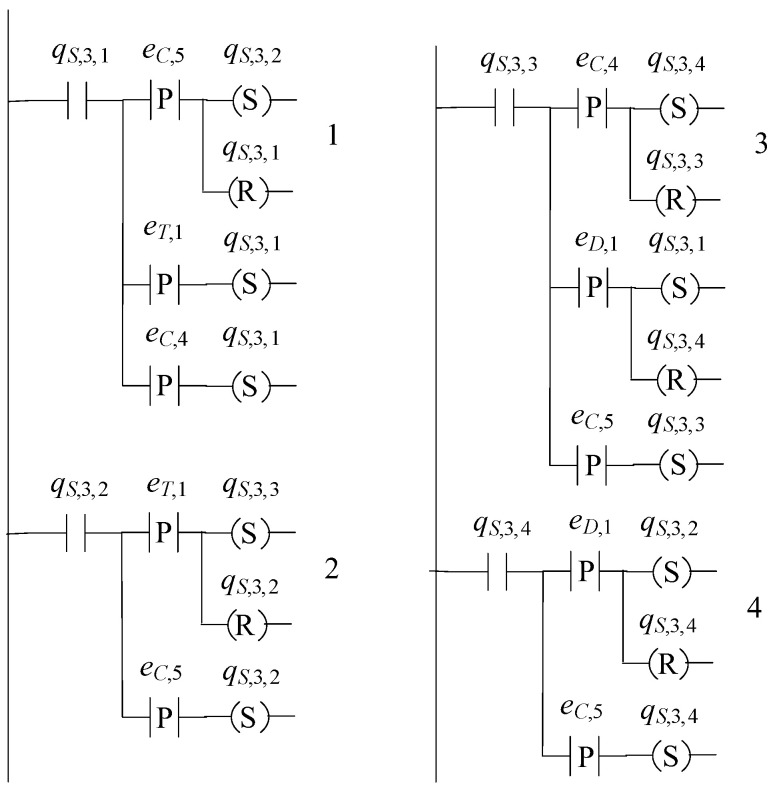
State diagram of $S_{3}$.

In Figure 12, Figure 13 and Figure 14, the supervisors realized in Section 4, are implemented using the IEC 61131-3 (2013) Ladder Diagrams. The implementation, through Ladder Diagrams, has been preferred, as the Ladder Diagrams provide a good overview and are offered for engineer inspection. Figure 12, Figure 13 and Figure 14, illustrate the ease implementability of the realized supervisors. As already mentioned in Section 4, the supervisors realized in the class of supervisors determined by $S_{1}$, are also offered for implementation in the event-driven architecture of the IEC 61499 function blocks. 

Regarding communication protocols, it is important to mention that the modern communication standard OPC UA as well as the Modbus protocol, being the typical PLC communication protocol, can be used through simple parametrization of the declared variables and the parameters of the default timers and the alarms of the PLC, see also [37]. Regarding further trends, imposed by Industry 4.0, see [33,37,38,39]. Finally, regarding the robotic manipulator, it is mentioned that the supervisor of the manipulator, implemented in PLC (see Figure 12), is interconnected to the robotic operations system (ROS2) following the directions presented in [40], providing an efficient framework.

## 8. Conclusions

In the present paper the model of manufacturing cell, in the presence of faults, has been developed through appropriate models of its subsystems. The DES models of all the system’s components have been presented considering possible actuator/sensor faults. The total automaton of the manufacturing cell has also been presented. The desired behavior of the manufacturing cell has firstly been presented analytically, in the form of nine desired specifications. The desired specifications have been translated to appropriate eleven prefixed closed regular languages. The desired languages have been determined from the eleven regular languages in combination to the marked behavior of the total system. The regular languages have been realized by a set of eleven supervisors. The supervisors have been developed upon realizing a two-state class of automata and two other automata. The supervisors have been designed to be as possible maximally permissive without losing necessary performance properties, while guaranteeing PR regarding the total automaton of the manufacturing cell. The performance of the controlled automaton has been proven to have satisfactory closed behavior and marked behavior. The controllability of the eleven proposed languages and the nonblocking property of the controlled automaton have been proven. The complexity of the proposed supervisory scheme has been computed. Finally, implementability issues to modern industrial control devices have been figured out and the ladder diagrams of the three automata classes have been developed. 

The feasibility of the results of the paper lies on two directions. The first direction is that the present supervisory control design is developed for a well-established and fully experimentally tested manufacturing cell with several applications, indicatively see [1,3,11]. The second is the implementation of the proposed supervisor scheme using Ladder diagrams (see Section 7).

The extension of the present supervisory control scheme, achieving tolerance to upper-level faults of a manufacturing process, to the case of partially observable lower-level faults in the devices of the process is currently under investigation.

## Figures and Tables

**Table 1:** States of the circular rotating table.

Symbol	State Description
$q_{T,1}$	The table is idle
$q_{T,2}$	The table is moving
$q_{T,3}$	The table is in faulty mode

**Table 2:** Events of the circular rotating table.

Symbol	Event Description
$e_{T,1}$	The table starts rotating for $90^{o}$
$e_{T,2}$	The table stops rotating
$e_{T,3}$	A fault took place
$e_{T,4}$	The fault has been repaired

**Table 3:** States of the classifier and transportation device.

Symbol	State Description
$q_{C,1}$	The device is idle
$q_{C,2}$	The device is classifying
$q_{C,3}$	The device has been paused
$q_{C,4}$	The device is transporting
$q_{C,5}$	The device is in faulty mode

**Table 4:** Events of the classifier and transportation device.

Symbol	Event Description
$e_{C,1}$	The device starts classifying
$e_{C,2}$	The product has been classified and accepted
$e_{C,3}$	The product has been classified and rejected
$e_{C,4}$	The device starts transporting.
$e_{C,5}$	The product has been transported.
$e_{C,6}$	A fault took place at the device
$e_{C,7}$	A fault has been repaired at the device

**Table 5:** States of the drilling machine.

Symbol	State Description
$q_{D,1}$	The drilling machine is idle
$q_{D,2}$	The drilling machine is working (drilling)
$q_{D,3}$	The drill is in faulty mode

**Table 6:** Events of the drilling machine.

Symbol	Event Description
$e_{D,1}$	The drilling machine starts drilling.
$e_{D,2}$	Drilling has been successfully completed
$e_{D,3}$	The machine is in faulty mode
$e_{D,4}$	The machine has been repaired.

**Table 7:** States of the testing device.

Symbol	State Description
$q_{B,1}$	The testing device is idle
$q_{B,2}$	The testing device is working
$q_{B,3}$	The testing device is in faulty mode

**Table 8:** Events of the testing device.

Symbol	State Description
$e_{B,1}$	The device begins the testing process *A*
$e_{B,2}$	The device begins the testing process *B*
$e_{B,3}$	The product is tested
$e_{B,4}$	A fault of the device took place
$e_{B,5}$	A fault of the device has been repaired

**Table 9:** States of the robotic manipulator.

Symbol	State Description
$q_{R,1}$	The manipulator is idle
$q_{R,2}$	The manipulator is retrieving a product from the table
$q_{R,3}$	The manipulator is storing a product
$q_{R,4}$	The manipulator is in faulty mode

**Table 10:** Events of the robotic manipulator.

Symbol	Event Description
$e_{R,1}$	The manipulator starts retrieving and storing a product.
$e_{R,2}$	The manipulator has retrieved a product from the table
$e_{R,3}$	The manipulator has stored a product
$e_{R,4}$	A fault of the manipulator took place
$e_{R,5}$	A fault of the manipulator has been repaired

**Table 11:** States of the feeding device.

Symbol	State Description
$q_{F,1}$	The device is idle
$q_{F,2}$	The device is working
$q_{F,3}$	The device is out of rough pieces
$q_{F,4}$	The device is in faulty mode

**Table 12:** Events of the feeding device.

Symbol	Event Description
$e_{F,1}$	The device starts working
$e_{F,2}$	A product has been fed
$e_{F,3}$	The device is out of rough products
$e_{F,4}$	The device has been refilled with rough products
$e_{F,5}$	A fault took place at the device.
$e_{F,6}$	A fault has been repaired at the device.

**Table 13:** Supervisors derived by $S_{1}$.

**Supervisor**	**Behavior**	Regular Expressions	Complexity
S11,1	K11	c11=eC,1+eD,1+eB,1+eR,1+eF,1+eT,4,c12=c13=eT,3,c14=eT,4	(2, 7, 9)
S11,2	K12	c11=eT,1+eC,1+eD,1+eB,1+eF,1+eR,5, c12=c13=eR,4,c14=eR,5	(2, 7, 9)
S21	K2	c11=ε,c12=c13=eC,5+eD,2+eD,3+eB,3, c14=eT,1	(2, 5, 9)
S31	K3	c11=eT,2+eT,4+eC,5+eC,6,c12=eT,1+eC,4,c13=ε,c14=c11	(2, 6, 10)
S41	K4	c11=eT,2+eT,4+eD,2+eD,4, c12=eT,1+eD,1,c13=ε,c14=c11.	(2, 6, 13)
S51	K5	c11=eT,2+eT,4+eB,3+eB,5, c12=eT,1+eB,1+eB,2,c13=ε,c14=c11	(2, 7, 11)
S61	K6	c11=eT,2+eT,4+eR,2+eR,5,c12=eT,1+eR,1,c13=ε,c14=c11	(2, 6, 12)
S81,1	K81	c11=eD,1, c12=c13=eD,2,c14=eB,1	(2, 3, 4)
S81,2	K82	c11=eD,1, c12=c13=eD,3,c14=eB,2	(2, 3, 4)

## Abbreviations and Nomenclature

The capital bold letter G, with appropriate indices, is used to denote manufacturing subsystems in DES form. The capital bold letter S, with appropriate indices, is used to denote supervisor automata in DES form. Finally, the capital letter K, with appropriate indices, is used to denote regular languages. In the following table, the acronyms used in the present paper are presented.

Acronyms

DES	Discrete Event System
SCT	Supervisory Control Theory
FMS	Flexible Manufacturing System
CNC	Computer Numerical Control
PLC	Programmable Logic Controller
IEC	International Electrotechnical Commission
C&T	Classifier and Transportation
SCADA	Supervisory Control and Data Acquisition
PR	Physical Realizability

A list including the symbols, used in the paper, is presented in the following table.

List of symbols.

$G_{\phi }$	The automaton of the subsystem indexed by ϕ, where ϕ is considered to be an appropriate capital letter representing the subsystem
${\mathbb{Q}}_{\phi }$ $,\;q_{\phi ,j}$	The set of states of $G_{\phi }$, the j-th state of $G_{\phi }$
$E_{\phi }$ $,\;E_{\phi ,uc}$ $,\;e_{\phi ,j}$	The alphabet of $G_{\phi }$, the uncontrollable events set of $G_{\phi }$, the j-th event of $G_{\phi }$
${ℍ}_{\phi }(q)$ $,\;f_{\phi }$	The active event set of the state q of $G_{\phi }$, the transition function of $G_{\phi }$
$x_{\phi ,0}$ $,\;{\mathbb{Q}}_{\phi ,m}$	The initial state of $G_{\phi }$, the set of the marked states of $G_{\phi }$
$L(G_{\phi })$ $,\;L_{m}(G_{\phi })$	The closed and the marked behavior of $G_{\phi }$
$G,\;G_{c}$	The total automaton, the total controlled automaton
Kν , KνD	The regular language of the ν-th specification and the respective desired language
Kij , KiD,j	The j-th regular language of the i-th specification and the respective desired language
EiS,j, EνS,	The alphabet of Kij, the alphabet of Kν
EiS,j,uc , EνS,uc	The uncontrollable event subsets of EiS,j and EνS, respectively.
Pij , Pν	The projections of $E^{*}$ to EiS,j* and EνS*, respectively.
Si1,j, Sν1	The supervisor automaton of the j-th regular language of the i specification, the supervisor automaton of the ν-th specification
ℚiS,1,j , ℚνS,1	The set of states of Si1,j, the set of states of Sν1
qiS,1,j,k , qνS,1,k	The k-th state of Si1,j, the k-th state of Sν1
ℍiS,1,j(q) , ℍνS,1(q)	The active event set of the state q of Si1,j and Sν1, respectively
fiS,1,j(q,e) ,fνS,1(q,e)	The transition functions of Si1,j and Sν1, respectively
xiS,1,j,0 , xνS,1,0	The initial state of Si1,j and Sν1, respectively
ℚiS,1,j,m , ℚνS,1,m	The set of the marked states of Si1,j, the set of the marked states of Sν1
$S_{\lambda }$	The supervisor automaton of K9 for λ=2 and of K7 for λ=3
${\mathbb{Q}}_{S,\lambda }$ $,\;q_{S,\lambda ,j}$	The set of states of $S_{\lambda }$, the j-th state of $S_{\lambda }$
${ℍ}_{S,\lambda }(q)$ $,\;f_{S,\lambda }(q,e)$	The active event set of the state q of $S_{\lambda }$, the transition function of $S_{\lambda }$
$x_{S,\lambda ,0}$ ,ℚiS,λ,m	The initial state of $S_{\lambda }$, the set of the marked states of $S_{\lambda }$
